# Beyond seasoning nutrients bioactive ingredients and healthcare effects of Allium vegetables

**DOI:** 10.3389/fnut.2025.1597788

**Published:** 2025-12-05

**Authors:** Hafiz Ghulam Muhu Din Ahmed, Jiazhen Yang, Tong Zhu, Rashid Iqbal, Qing Sheng, Ruijie Dong, Wenhuai Tian, Lei Xiao, Yawen Zeng, Yumei Ding

**Affiliations:** 1 Department of Plant Breeding and Genetics, Faculty of Agriculture and Environment, The Islamia University of Bahawalpur, Bahawalpur, Pakistan; 2 Biotechnology and Germplasm Resources Institute, Yunnan Academy of Agricultural Sciences, Kunming, China; 3 College of Food Science and Technology, Yunnan Agricultural University, Kunming, China; 4 Department of Agronomy, Faculty of Agriculture and Environment, The Islamia University of Bahawalpur, Bahawalpur, Pakistan; 5 Department of Life Sciences, Western Caspian University, Baku, Azerbaijan

**Keywords:** Allium vegetables, bioactive compounds, functional foods, preventive healthcare, bioavailability, antioxidants, organosulfur compounds, flavonoids

## Abstract

This review highlights the nutritional and therapeutic significance of Allium vegetables—including garlic, onion, leek, and chive—emphasizing their principal bioactive compounds such as organosulfur compounds, flavonoids, and essential micronutrients. These phytochemicals exhibit potent antioxidant, anti-inflammatory, and anticancer activities that contribute to the prevention and management of chronic diseases including cancer, diabetes, and cardiovascular disorders. Mechanistic studies indicate that Allium-derived compounds modulate oxidative stress and inflammatory pathways through NF-κB and Nrf2 signaling, thereby enhancing metabolic and immune resilience. Despite these well-established benefits, challenges such as low bioavailability, inter-species variability, and limited clinical validation restrict translational potential. Recent advances in nanoencapsulation, emulsion-based delivery, and inclusion complexes offer promising strategies to improve compound stability and absorption. Future research should integrate clinical validation, comparative genomics, and functional food formulation to maximize health outcomes. This review underscores Allium vegetables as promising functional foods for preventive healthcare and supports their integration into daily diets to promote sustainable wellbeing.

## 1 Introduction

Alliums, belonging to the Alliaceae family, encompass a diverse genus that includes garlic (*Allium sativum*), onions (*Allium cepa*), shallots (*Allium cepa var. aggregatum*) leeks (*Allium ampeloprasum*), and chives (*Allium schoenoprasum*). These plants are classified based on morphological traits and genetic diversity, with over 750 species identified globally, particularly in the Northern Hemisphere (1). Alliums are renowned for their culinary and medicinal uses, attributed to their rich content of bioactive compounds such as organosulfur compounds (OSCs), flavonoids, and polysaccharides, which confer antioxidant, antimicrobial, and anticancer properties (2). The classification of Allium species often involves both qualitative and quantitative morphological descriptors, which help elucidate taxonomic relationships and intra-species variability (3). Their significant role in traditional medicine and modern dietary practices highlights their importance in promoting health and preventing diseases. Bioactive constituents like OSCs, phenolic acids, and flavonoids contribute to potent antioxidant activity, demonstrated by DPPH and ABTS radical scavenging assays, as well as significant antimicrobial effects against *Escherichia coli*, *Staphylococcus aureus*, and *Salmonella enterica*. Pharmacological studies have also reported anti-inflammatory activity, linked to suppression of NF-κB activation and reduced pro-inflammatory cytokine production (4).

While numerous studies have explored the nutritional and pharmacological attributes of Allium species, comprehensive reviews integrating their mechanistic pathways, bioavailability challenges, and translational potential remain limited. This review bridges that gap by synthesizing recent advances in bioactive compound characterization, delivery systems, and preventive healthcare relevance, thereby offering a holistic understanding of Alliums as functional foods with clinical and dietary significance (2, 5).

**Figure 1:**
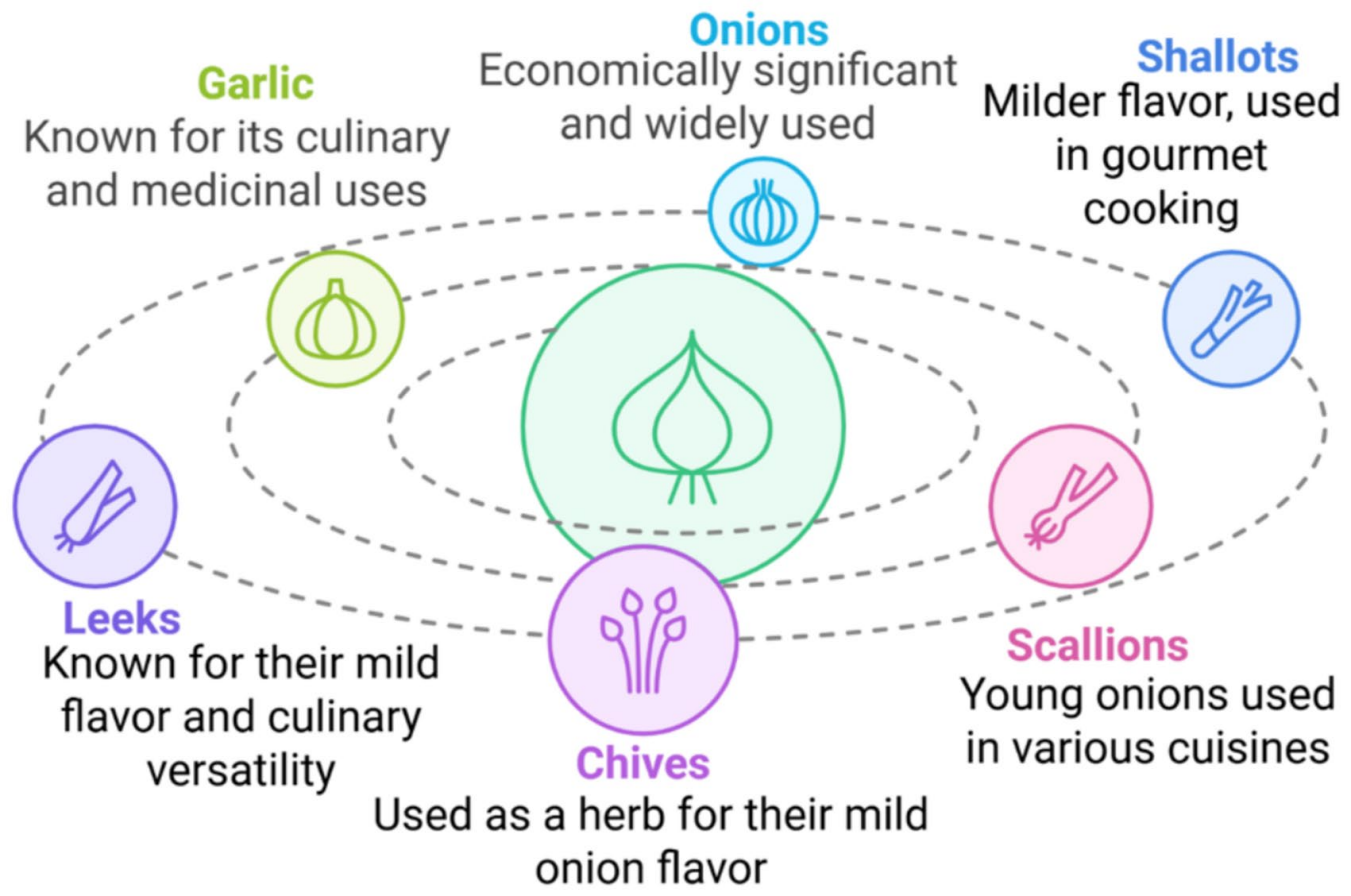
Phyto-nutritional relationships among major Allium species and their functional roles in health. The central circle represents the Allium genus, illustrating its shared phylogenetic and biochemical foundation, primarily characterized by sulfur-containing metabolites. Each outer circle denotes a key Allium species—garlic (*A. sativum*), onion (*A. cepa*), shallot (*A. cepa* var. *aggregatum*), leek (*A. ampeloprasum*), and chive (*A. schoenoprasum*)—with annotated connections highlighting their dominant bioactive compounds (e.g., allicin, quercetin, kaempferol) and principal applications. Arrows from the central circle to each outer segment indicate the species’ shared nutritional origins but diversified culinary uses (e.g., flavor enhancement, spice formulations) and therapeutic attributes (e.g., antimicrobial, cardioprotective, anticancer effects). This visualization underscores the evolutionary and functional continuum within the Allium genus linking biochemical diversity to human health outcomes.

Garlic and onions represent the economically most important members of the *Allium* genus; extensive cultivation for their uses, which are immense in quantity and species, is shown to grow in various climatic conditions as represented in Figure 1.

### 1.1 Historical significance and cultural relevance in various cuisines

Members of Allium species have significant repute in the annals of food and medicine. Garlic has been consumed since the time of ancient Mesopotamia and the Egyptian civilization for its culinary and medicinal uses (6, 7). It was found in King Tutankhamun’s tomb and was also provided to the laborers of Great Pyramids of Giza for stamina and to keep away various diseases (8, 9). The garlic and onions had an immense use during the Greco-Roman period amongst the soldiers and the athletes hence showing the capability of strength gain and stamina.

The cultural relevance of Alliums cuts across the globe. In Asia, for instance, garlic and onion have formed part of the traditional Chinese medicine for over several millennia because of their efficiency in treating respiratory and digestive disorders (10). In Asia, garlic is a vital component of Ayurvedic medicine, used to treat infections, high blood pressure, and digestive disorders, among other health conditions (11, 12). In Europe, the Alliums played a big role in the diet and medicine throughout the Middle Ages. Onions and garlic were eaten for antibacterial reasons; they were such a valuable item since they allowed the people a way to preserve and give flavor to food without refrigeration (9, 10).

The cultural and historical significance of Alliums is not confined to their usage in medicine but also includes vital roles in gastronomic traditions. In the Middle Eastern, Mediterranean, and Asian cuisines, garlic and onions, together with other Alliums, are indispensable. They were applied in many manners, as flavor enhancers in preparations ranging from simple stews to complex sauces (13, 14). For example, garlic was consumed as part of the Mediterranean diet and has been associated with a reduced risk of cardiovascular disease (8, 15). Equally, onions and garlic were at the core of flavor in Indian curries, French onion soup, and Spanish gazpacho, proving the global importance of plants of culinary significance (16, 17).

While numerous reviews have addressed the pharmacological properties of Allium species, few have comprehensively combined their nutritional value, mechanistic insights, and clinical relevance in a unified framework. This review aims to bridge that gap by critically evaluating not only the bioactive profiles and associated health benefits of Allium vegetables, but also their metabolism, bioavailability challenges, and translational potential (18). Compared to prior works, our review integrates historical, phytochemical, and functional data with current innovations in delivery systems, offering a holistic view of Allium’s therapeutic promise.

### 1.2 Global production and cultivation trends

To contextualize the global production and assess recent cultivation trends, a historical comparison of the major Allium crops is essential. Table 1 presents the global production volume of garlic, onions, and leeks/chives over the five-year period from 2018 to 2022, highlighting the consistent growth in production across the genus. This historical trend demonstrates a significant and consistent increase in the production of onions and leeks/chives (9.29 and 11.6% growth, respectively) between 2018 and 2022, underscoring their rising importance as staple and high-value crops. While garlic production has stabilized at a high level, showing a modest 2.15% increase over the period, the overall expansion in Allium cultivation signals a growing global accessibility of these nutritionally and medicinally valuable vegetables. These cultivation trends have positive implications for enhancing global nutritional security and the availability of their associated bioactive compounds. Garlic production has surged to approximately 29.15 million tonnes in 2022, with China alone responsible for roughly 73% of this output, followed by India (~3.2 million t) and Bangladesh. Meanwhile, onion and shallot production totaled about 5 million tonnes. China and India remain leading producers—India yielded ~31.7 million tonnes, China ~24.5 million t. Other important Allium crops include leeks, with over 2 million tonnes produced globally in 2022 (notably Spain and Germany), and green onions, cultivated extensively in East Asia—China alone accounts for more than 500,000 ha of production. These trends underscore the agricultural and nutritional significance of Alliums worldwide.

**Table 1:** Global production of major *Allium* species (2018–2022).

Crop	2018 (Million Tonnes)	2019 (Million Tonnes)	2020 (Million Tonnes)	2021 (Million Tonnes)	2022 (Million Tonnes)	5-year change (2018–2022)
Garlic	28.53	29.56	28.16	29.28	29.15	↑ 2.15%
Onions (Dry)	102.39	107.56	107.72	109.91	111.9	↑ 9.29%
Leeks & Other (*incl. chives*)	2.5	2.52	2.65	2.76	2.79	↑ 11.6%$

Food and Agriculture Organization of the United Nations (FAO). FAOSTAT Database. [Crops and livestock products statistics]. Rome, Italy. Accessed [Date of Access, e.g., 24 October 2025]. Available at: https://www.fao.org/faostat/en/#data/QCL.

### 1.3 Nutritional and functional composition of Allium vegetables

Allium species, including onions (*Allium cepa* L.) and garlic (*Allium sativum* L.), are rich in both macronutrients and micronutrients. Mainly, the composition included carbohydrates, proteins, and essential oils, with garlic containing about 29% carbohydrate and 6.3% protein (19). Onions were good sources of ascorbic acid, phosphorus (P), and calcium (Ca) (20). Other micronutrients present, though there is great variation between varieties, some of which were relatively rich in these elements, include zinc, manganese, and selenium (21). Bioactive substances in foods that have health benefits apart from basic nutrition have therefore become important substances in preventive health care. These components of functional foods include the following: carotenoids, dietary fiber, flavonoids, and omega fatty acids, which could prevent chronic diseases like cancer, cardiovascular diseases, and type II diabetes (3, 22). The awareness regarding the diet-health relationship has increased, raising demand for functional foods in improving human health and preventing a variety of diseases due to their proven potential (23, 24). Dietary variety is believed to ensure adequate intake, with bioactive compounds potentially being sourced from both plants and animals. Therefore, adding functional components into everyday nutrition will make a great difference for public health by offering nutritional deficiencies and the resistance of diseases.

Comparative nutritional composition of Allium vegetables presented in Table 2. Garlic has a higher caloric content than other Allium vegetables, providing 149 kcal per 100 g, yet it remains relatively low in calories compared to many staple foods. Onions are low in energy value −40 kcal but high in quercetin and sugars (16, 17). Leeks contain moderate calories (61 kcal), significant folate [16% (daily value) DV], and sulfur compounds. Chives are nutrient-dense, high in vitamin C (58.1 mg, 97% DV), calcium (83 mg), and folate (26% DV), which helps in immunity and bone health. Shallots balance out moderate calories (72 kcal) against OSCs and flavonoids, which enhance antioxidant and metabolic benefits across Allium vegetables (25).

**Table 2:** Comparative overview of the nutritional contents in Alliums vegetables.

Nutrient	Garlic (100 g)	Onion (100 g)	Leeks (100 g)	Chives (100 g)	Shallots (100 g)
Calories (kcal)	149	40	61	30	72
Protein (g)	6.4	1.1	1.5	3.3	2.5
Fat (g)	0.5	0.1	0.3	0.7	0.1
Carbohydrates (g)	33.1	9.3	14.2	5.1	16.8
Fiber (g)	2.1	1.7	1.8	2.5	2.1
Sugars (g)	1	4.2	3.9	1.9	6.7
Vitamin C (mg; % DV)	31.2 (52%)	8.1 (13%)	12.2 (20%)	58.1 (97%)	6.7 (11%)
Vitamin B6 (mg; % DV)	1.2 (15%)	0.1 (6%)	0.2 (10%)	0.2 (15%)	0.1 (6%)
Folate (μg; % DV)	3 (11%)	19 (14%)	64 (16%)	105 (26%)	34 (9%)
Calcium (mg; % DV)	181 (18%)	23 (2%)	59 (6%)	83 (8%)	37 (4%)
Iron (mg; % DV)	1.7 (9%)	0.2 (1%)	1.5 (8%)	1.1 (6%)	1.2 (7%)
Potassium (mg; % DV)	401	146	180	296	334
Manganese (mg; % DV)	1.7 (85%)	0.1 (5%)	0.2 (10%)	0.2 (10%)	0.2 (10%)
Bioactive compounds	Allicin, OSCs (e.g., diallyl disulfide)	Quercetin, OSCs (e.g., diallyl disulfide)	OSCs, Flavonoids	Vitamin C, OSCs, Flavonoids	OSCs, Flavonoids

DV, Daily Value. All data in this table are supported by references cited in the main text.

Nutrient composition varies among these species: leeks are notable for their high content of vitamin K, folate, and the flavonoid kaempferol; chives are particularly rich in vitamin C, vitamin A (as β-carotene), and lutein; shallots have a milder flavor profile due to lower allicin content but provide appreciable levels of OSCs such as methyl propyl disulfide; scallions are good sources of vitamin C, vitamin K, and small amounts of quercetin; and garlic chives contain abundant carotenoids, vitamin C, and saponins (16, 17).

#### 1.3.1 Overview of vitamins (vitamins C, B6)

Allium vegetables, represented here by garlic, onion, and leeks, due to their very richness in a wide range of vitamins and essential minerals, have an immense effect on human health. Notably, these vegetables are a good source of ascorbic acid, which ranges from 9.7 to 15.6 mg/100 g in garlic and vitamin B6, whose concentration as high as 2.04 mg/100 g has been reported for some varieties of garlic (14). Besides, they supply vital minerals such as potassium, calcium, magnesium, and manganese, serving an array of physiologic functions (18, 25–29). Vitamin C is highly essential in collagen synthesis, wound healing, and in preventing oxidative stress. Other than that, the Alliums also contain vitamin B6, which is highly essential in the development of the brain and the development of the immune system, and for amino acid metabolism (21, 30).

#### 1.3.2 Minerals (manganese, selenium) in Alliums

Minerals such as phosphorus (P), potassium (K), calcium (Ca), magnesium (Mg), zinc (Zn), manganese (Mn), sodium (Na), iron (Fe), bromine (Br), iodine (I), selenium (Se), and copper (Cu), in addition to vitamins A, C, B6, and folate, are present in Allium species. Besides that, manganese and selenium minerals are highly abundant in Alliums. Manganese is responsible for bone formation, blood clotting, and reduction of oxidative stress (13, 31). Though manganese and selenium equally contribute to antioxidant activity by the neutralization of free radicals, reducing oxidative stress. Selenium is an essential trace metal with antioxidant properties that actively participates in thyroid function, DNA (deoxyribonucleic acid) synthesis, and protection against oxidative damage and infection (32). These minerals have a shielding effect on the cell and facilitate many enzyme systems of the body.

#### 1.3.3 Macronutrients and micronutrients in Alliums

Alliums form part of the important sources of major and minor nutrients; hence, they are part of a healthy diet. For example, garlic contains approximately 33% carbohydrates and 5–6% protein by dry weight, while being low in fat at around 0.5% (21, 25). Similarly, onions contain about 9% carbohydrates, 1.1% protein, and minimal fat, making them low-calorie but nutrient-dense vegetables (12, 19). The micronutrient profile of Allium is astonishingly attractive. Macronutrients Mineral substances like nitrogen, phosphorus, and potassium were directly associated with growth and quality in crops of this nature; their fertilization has a direct impact on yield (33). Furthermore, garlic chives contain different quantities of mineral substances depending on the plant age; in general, the older plants contain more nitrogen and phosphorus (2, 19).

#### 1.3.4 Caloric content and common dietary uses

**Figure 2:**
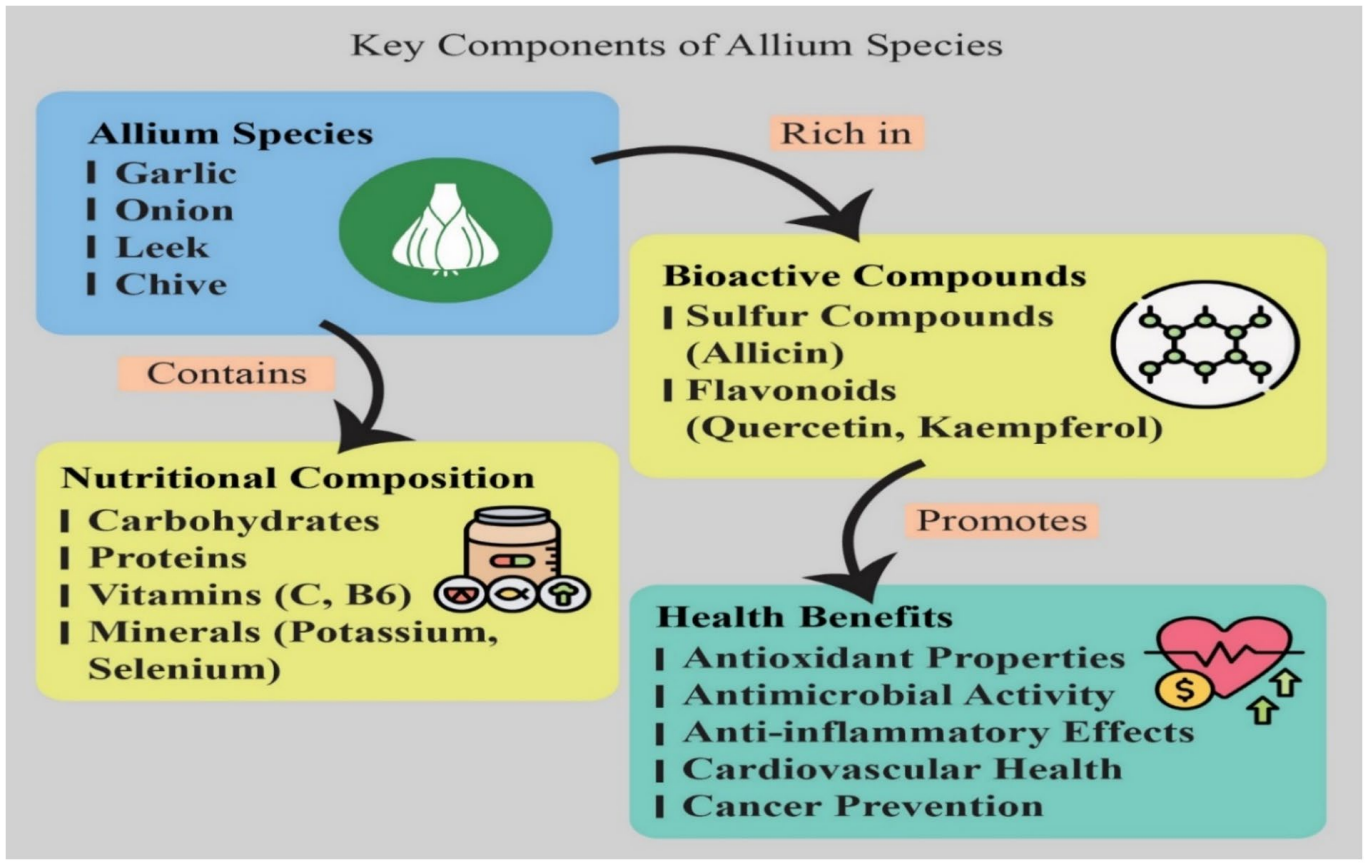
Key components, nutritional composition, and associated health benefits of Allium species. This diagram illustrates the main components and health-promoting effects of common Allium species. The Blue Box identifies key members of the Allium genus, including Garlic, Onion, Leek, and Chive. These species are categorized by two major compositional pathways. 1. Nutritional Composition (Yellow-Orange Box): Allium species inherently contain a substantial Nutritional Composition, including macronutrients such as Carbohydrates and Proteins. They are also rich sources of essential micronutrients, specifically Vitamins (e.g., Vitamin C and B6) and Minerals (e.g., Potassium and Selenium). 2. Bioactive Compounds (Yellow Box): The species are particularly rich in various Bioactive Compounds, which are responsible for many of their distinctive properties and pungent flavors. Key among these are Sulfur Compounds, such as Allicin (a thiosulfinate often studied in garlic), and Flavonoids (a type of polyphenol), including Quercetin and Kaempferol. A chemical structure of a sulfur-containing compound is included for visual context. 3. Health Benefits (Green Box): These components collectively promote significant Health Benefits. These include: Antioxidant Properties: Due to high flavonoid and sulfur compound content, which helps neutralize free radicals. Antimicrobial Activity: Primarily attributed to sulfur compounds like allicin, which can inhibit the growth of various bacteria, fungi, and viruses. Anti-inflammatory Effects: Contributing to the management of chronic inflammatory conditions. Cardiovascular Health: Associated with effects like blood pressure and cholesterol regulation. Cancer Prevention: Linked to the inhibitory actions of bioactive compounds on carcinogen activation and tumor cell proliferation.

Allium vegetables, including onions, garlic, leeks, and chives, are generally low in calories, with garlic being the most calorie-rich among them. Among the major benefits of Alliums, it is necessary to outline that they possess very low caloric value and can be used in a great number of diets. Thus, 100 g of raw garlic contain about 149 kcal, while 100 g of raw onions have a value of about 40 kcal (25). This low calorific content allows the inclusion of Alliums in many dishes without piling excess calories within them, which is a concern for people looking to manage body weight (9, 19, 21). Nutritional and phytochemical profiles of Allium vegetables contribute highly due to their rich bioactive profile to their dietary uses and health benefits as depicted in Figure 2. These vegetables contain OSCs, flavonoids, and phenolic compounds along with vitamins, associated with a wide range of health-related properties, which include the prevention of oxidative stress, inflammation, and carcinogenesis (34, 35).

In addition to their culinary appeal, Alliums are also valued for their health benefits. In spite of their highly negligible caloric value, Alliums add a great depth of flavor to dishes, which dispenses with adding any high-fat ingredient or immense seasoning with salt. Most of them serve as seasonings in soups, stews, sauces, and salads, eaten raw, cooked, or fermented. Most culinary traditions cannot do without Alliums in foundational cooking techniques like sautéing or braising, wherein layers of flavor are determined. Their ability to stimulate appetite and be healthy at the same time makes them irreplaceable in cuisines all over the world (36–38).

#### 1.3.5 Functional components and their relevance in preventive healthcare

The preventive healthcare, through the action of functional components in some Allium species on a wide variety of chronic diseases, is highly underlined, especially the OSCs, flavonoids, and saponins. Such bioactive compounds have multiple health properties, including antioxidant, anti-inflammatory, and antimicrobial activities important in such conditions as metabolic syndrome, cardiovascular diseases, and diabetes (8, 39). OSCs, for instance, have been reported to lower blood pressure and cholesterol levels, while flavonoids like quercetin also take part in the anti-cancerous action of garlic. The various phytochemicals in Allium species, apart from improving their nutritional value, support the development of functional foods and nutraceuticals aimed at chronic disease prevention, in line with their relevance in modern dietetic practice. Thus, integrating Allium into common foods will bring immense health benefits in human beings and reduce disease burdens (40, 41).

## 2 Bioactive compounds in Alliums

### 2.1 Organosulfur compounds (OSCs)

**Figure 3:**
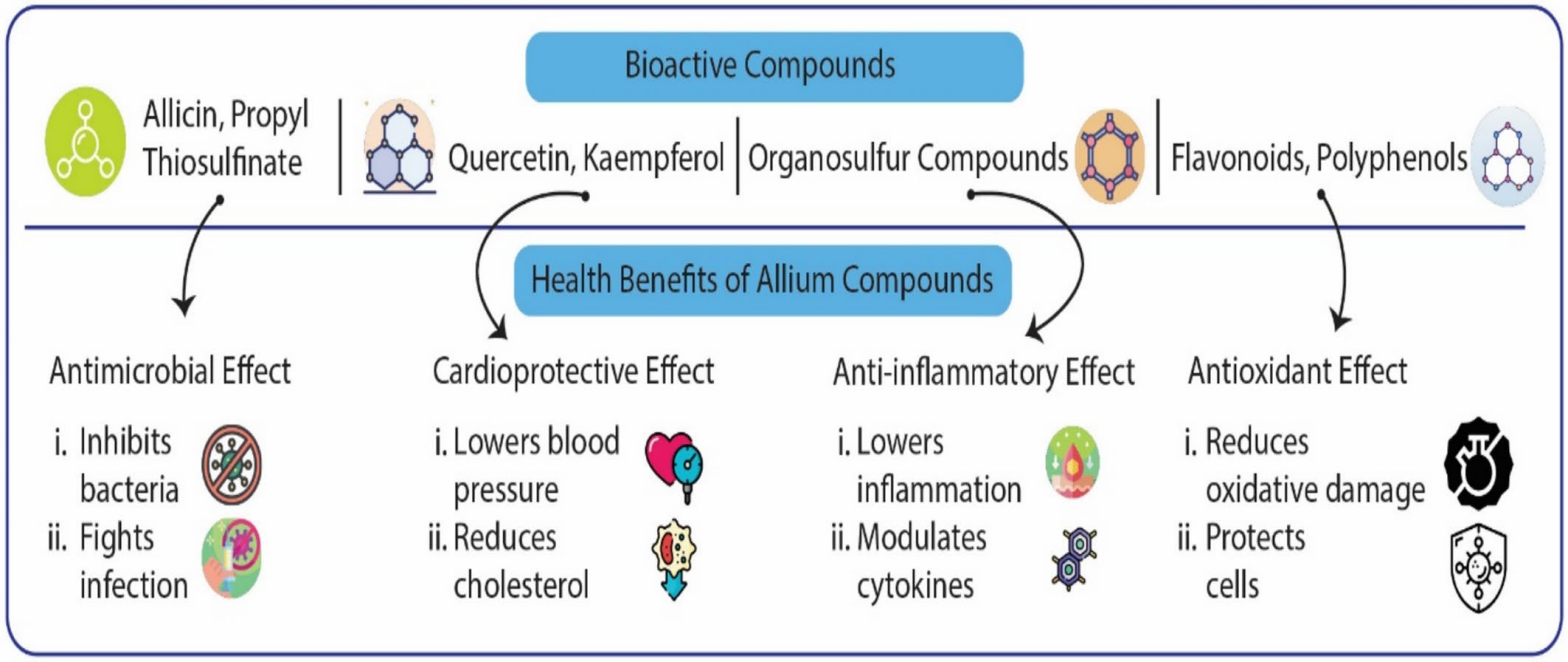
Specific bioactive compounds from Allium species and their corresponding pharmacological effects. This diagram illustrates the relationship between four major categories of bioactive compounds found in the Allium genus and their resultant health benefits. The top bar identifies the Bioactive Compounds, which include Allicin and Propyl Thiosulfinate (sulfur compounds), Quercetin and Kaempferol (flavonoids), other general Organosulfur Compounds, and various Flavonoids/Polyphenols. The bottom bar delineates the corresponding Health Benefits attributed to these compounds: Antimicrobial Effect: Primarily mediated by Allicin and Propyl Thiosulfinate, this effect involves the inhibition of bacteria and a general capacity to fight infection. Cardioprotective Effect: Linked to Quercetin and Kaempferol, this benefit includes the ability to lower blood pressure and reduce cholesterol levels, supporting overall heart health. Anti-inflammatory Effect: Attributed broadly to Organosulfur Compounds, this action works by lowering inflammation and modulating pro-inflammatory cytokines to restore balance. Antioxidant Effect: Driven by the collective action of Flavonoids and Polyphenols, this key function involves reducing oxidative damage from free radicals and thus protecting cells against degradation and disease.

One of the most well-researched OSCs in Alliums is allicin, a compound primarily found in garlic. Allicin is produced enzymatically from alliin when garlic cloves are crushed or chopped. The enzyme alliinase converts alliin to allicin, which gives garlic its characteristic pungent aroma and is responsible for many of its therapeutic properties (40, 42). However, allicin is highly unstable and quickly degrades into other organosulfur-containing compounds such as diallyl disulfide and diallyl trisulfide, which also contribute to garlic’s bioactivity (12, 43). The anti-inflammatory effects therefore place allicin as a potential therapeutic agent in inflammatory conditions. Bioactive compounds and their effects on human health illustrated in Figure 3.

#### 2.1.1 Mechanistic action and stability of allicin

Allicin’s primary mode of action is its ability to rapidly undergo thiol-disulfide exchange with free sulfhydryl (SH) groups on cysteine residues of vital proteins and enzymes. This process, known as S-thiolation, effectively modifies the function of key cellular targets. Examples include:

*Antimicrobial Action:* Allicin irreversibly inhibits essential thiol-containing enzymes (e.g., alcohol dehydrogenase) in bacterial and fungal pathogens (3, 44).

*Anti-inflammatory Effects:* Allicin and its breakdown products react with the cysteine residues of KEAP1, leading to its dissociation from Nrf2 and subsequent activation of the antioxidant response element ARE pathway. Simultaneously, it can suppress the pro-inflammatory NF-B signaling cascade. Due to its high reactivity, allicin is highly unstable, possessing a short half-life (measured in minutes) at physiological pH and temperature. Consequently, its systemic concentration is low. It rapidly breaks down into more stable, lipid-soluble secondary metabolites (45–47).

#### 2.1.2 Other sulfur compounds (e.g., Diallyl sulfides, thiosulfates) and their biological activities

Apart from allicin, there are many other bioactive sulfur compounds present in garlic and other Alliums. For example, when the amino acid allicin breaks down it forms diallyl sulfides. Diallyl sulfides have been proven to have anticarcinogenic effects as well as cardioprotective effects. It is hypothesized that through inducing apoptosis (programmed cell death) in cancerous cells the diaillyl sulfides inhibit carcinogenesis and modulate systems of enzymes that detoxify carcinogens (48, 49). Thiosulfates are other categories of sulfur derivatives occurring in Allium spp., which have been attributed to both antimicrobial and antioxidant activities as well. Further established various cardiovascular benefits through the improvement in the lipid profile and reduction in blood pressure levels (25, 40). Studies have shown that increasing the number of sulfur atoms (e.g., from mono- to di- to tri-sulfides) enhances redox reactivity by improving electron delocalization and radical stabilization, which enhances their free-radical scavenging potential. For instance, diallyl trisulfide (DATS) exhibits stronger antioxidant activity than DADS (Diallyl disulfide) or diallyl sulfide (DAS) due to the additional sulfur atom improving redox potential and increasing lipophilicity, which facilitates membrane penetration and intracellular activity. Additionally, the presence of alk(en)yl groups contributes to the compound’s electron-donating capacity and hydrophobicity, further enhancing interaction with lipid bilayers and scavenging of lipid peroxyl radicals (50, 51).

### 2.2 Flavonoids and phenolic compounds

#### 2.2.1 Types of flavonoids found in Alliums (e.g., quercetin, kaempferol) and their antioxidant properties

**Figure 4:**
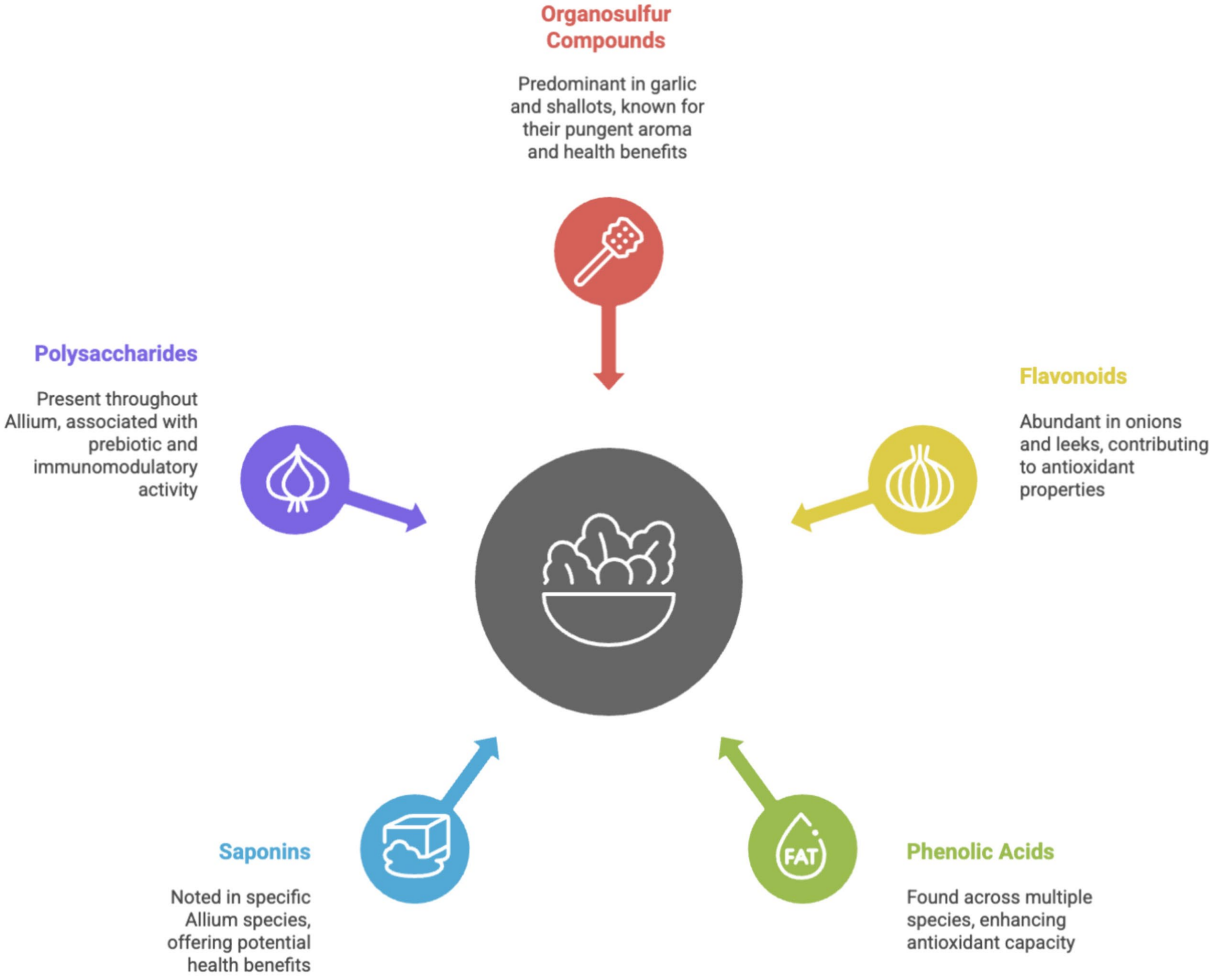
Phytochemical profile of Allium vegetables. Allium vegetables, encompassing garlic, onions, leeks, and shallots, are rich sources of diverse phytochemicals that underpin their recognized culinary and medicinal value. The predominant active constituents are organosulfur compounds, particularly abundant in garlic and shallots. These compounds, responsible for the characteristic pungent aroma and flavor, yield crucial health benefits, including antimicrobial, antioxidant, and cardioprotective properties. Beyond organosulfur compounds, the Allium genus is a significant source of polyphenols. This class includes flavonoids, notably quercetin prevalent in onions and leeks, which contribute strong antioxidant capacity and potential anti-inflammatory effects. Furthermore, phenolic acids are found across multiple Allium species, collectively enhancing the vegetable’s overall antioxidant defense mechanisms. Structurally distinct from these are saponins, noted in specific species, offering potential benefits such as cholesterol reduction and immunomodulatory activity. Finally, polysaccharides, mainly fructans like inulin, are present throughout the bulb, functioning as prebiotics that support beneficial gut flora and contribute to overall gut health. Collectively, this intricate profile of phytochemicals highlights the multi-faceted contribution of Allium consumption to human health and disease prevention.

Among the most prevailing flavonoids in Allium vegetables, onions have been found to have quercetin, kaempferol, and a few derivatives of quercetin, which contribute to their antioxidant activity (52, 53). For example, different studies report that as high as 36.94% of the flavonoids in onion extracts are quercetin. Antioxidant activities of Allium extracts were quantitatively measured by methods such as the DPPH (2,2-diphenyl-1-picrylhydrazyl) and ABTS (2,2′-azino-bis(3-ethylbenzothiazoline-6-sulfonic acid)) assays, which showed high scavenging activities and were positively correlated to flavonoid content in these plants (54–56). In particular, quercetin has evidenced the scavenging of free radicals and influenced cellular signaling pathways that enhance its protective effects against oxidative damage (57). Due to the different flavonoid mechanisms like modulation of many key cellular activities, flavonoids have been mentioned as one of the most potent candidates for therapeutic agents in functional foods. Incorporation of dietary sources of bioactive molecules will vastly improve the health status. Amongst the different flavonoids, quercetin is abundant in Alliums, along with a few others like kaempferol. Quercetin and kaempferol are two phytochemicals that have anti-diabetic action through increasing insulin sensitivity and decreasing blood sugar levels as shown in Figure 4.

#### 2.2.2 Phenolic acids

The other classes of bioactive compounds in Alliums are the phenolic acids which are also antioxidants. These phenolic compounds include caffeic acid, ferulic acid, and p-coumaric acid reported to prevent initiation of oxidative stress and to prevent lipid peroxidation so important in protection against oxidative-related damage that is associated with aging and chronic diseases (33, 36). Various biological activities of phenolic acids are responsible for the health benefits provided by the vegetables belonging to the genus Allium (30, 48, 58). In addition, the bioactive phytochemicals in Allium vegetables, especially allicin and flavonoids, enhance antioxidant defense and impede tumor development by cell cycle arrest or suppression of angiogenesis (26).

### 2.3 Advanced delivery systems for enhancing bioavailability

One of the key limitations in harnessing the full therapeutic potential of Allium bioactive components, such as allicin, diallyl disulfide (DADS), S-allyl cysteine (SAC), and quercetin, is their low stability and poor bioavailability. Liposome-based delivery systems offer another promising approach, providing a phospholipid bilayer that shields sensitive OSCs from gastric acid while facilitating cellular uptake (14, 36). Emulsion-based systems, including nanoemulsions, have been successfully employed to increase the solubility and sustained release of hydrophobic flavonoids, such as quercetin, thereby prolonging their antioxidant activity in the body. Several *in vitro* and *in vivo* studies have demonstrated significant improvements in the plasma concentration and bioactivity of Allium-derived compounds through these systems, with some formulations progressing toward functional food or nutraceutical applications.

Similarly, liposomal formulations of quercetin from onion extracts exhibited enhanced intestinal permeability and prolonged plasma half-life in *in vivo* rat models compared to free quercetin. Among tested systems, polymeric nanoparticles and nano-emulsions have shown superior scalability and compatibility with food matrices, offering potential for functional food applications. However, challenges persist regarding industrial-scale production, cost-effectiveness, and compound–matrix interactions, which may influence encapsulation efficiency. Future work should integrate comparative evaluations of delivery systems across diverse Allium compounds to optimize formulation design, targeting bioavailability without compromising sensory or nutritional quality. These delivery innovations hold potential not only to overcome bioavailability barriers but also to broaden the scope of Allium-based interventions in disease prevention and health promotion (35, 55, 56).

## 3 Health benefits of Alliums

Recent research has highlighted the significant health benefits of Allium vegetables, such as garlic and onions, across cell, animal, and human models, with a focus on their bioactive compounds and underlying mechanisms. Allium species, are rich in sulfur-containing compounds, flavonoids, and saponins, which contribute to their diverse pharmacological actions, including antioxidant, anticancer, anti-inflammatory, and cardio-protective effects (51). Garlic, in particular, has been shown to exert protective effects on the vascular system by enhancing endothelial function, reducing platelet aggregation, and inhibiting lipid peroxidation, which are beneficial in managing conditions like atherosclerosis and diabetes (27). Key compounds include OSCs such as allicin, diallyl sulfides, and S-allylmercaptocysteine, which exhibit potent antioxidant, anticancer, and anti-inflammatory properties. Flavonoids like quercetin and kaempferol found in onions also play a crucial role in neutralizing oxidative stress and enhancing cardiovascular health (14, 51). These compounds have been shown to induce apoptosis, inhibit tumor proliferation, and improve metabolic disorders. However, challenges remain regarding their bioavailability and the need for innovative extraction methods to maximize their therapeutic potential (28). Overall, the multifaceted health benefits of Allium vegetables underscore their potential as functional foods and nutraceuticals in disease prevention and management.

### 3.1 Antioxidant properties

Diseases can be prevented by antioxidants in Alliums through their huge content of bioactive compounds, including OSCs, flavonoids, and polyphenols. These compounds also expressed remarkable antioxidant activities through scavenging free radicals, thus mitigating oxidative stress and minimizing the process of various diseases including cancer and cardiovascular conditions (29) as mentioned in Table 2. For example, it was pointed out that allicin and flavanol compounds have properties that induce apoptosis of cancer cells and act against tumor proliferation. Moreover, the Allium species has been capable of demonstrating immune-enhanced capabilities in enhancing the body’s immune responses to disease or infection. This is further corroborated by *in vitro* and in silico antioxidant activity of the Alliums in preventing oxidative damage and generally contributing positively to health (59, 60). Thus, dietary inclusion of Alliums is likely to confer protection against chronic diseases based on their multi-drug antioxidant mechanisms.

#### 3.1.1 Mechanisms of action for antioxidants in disease prevention

**Figure 5:**
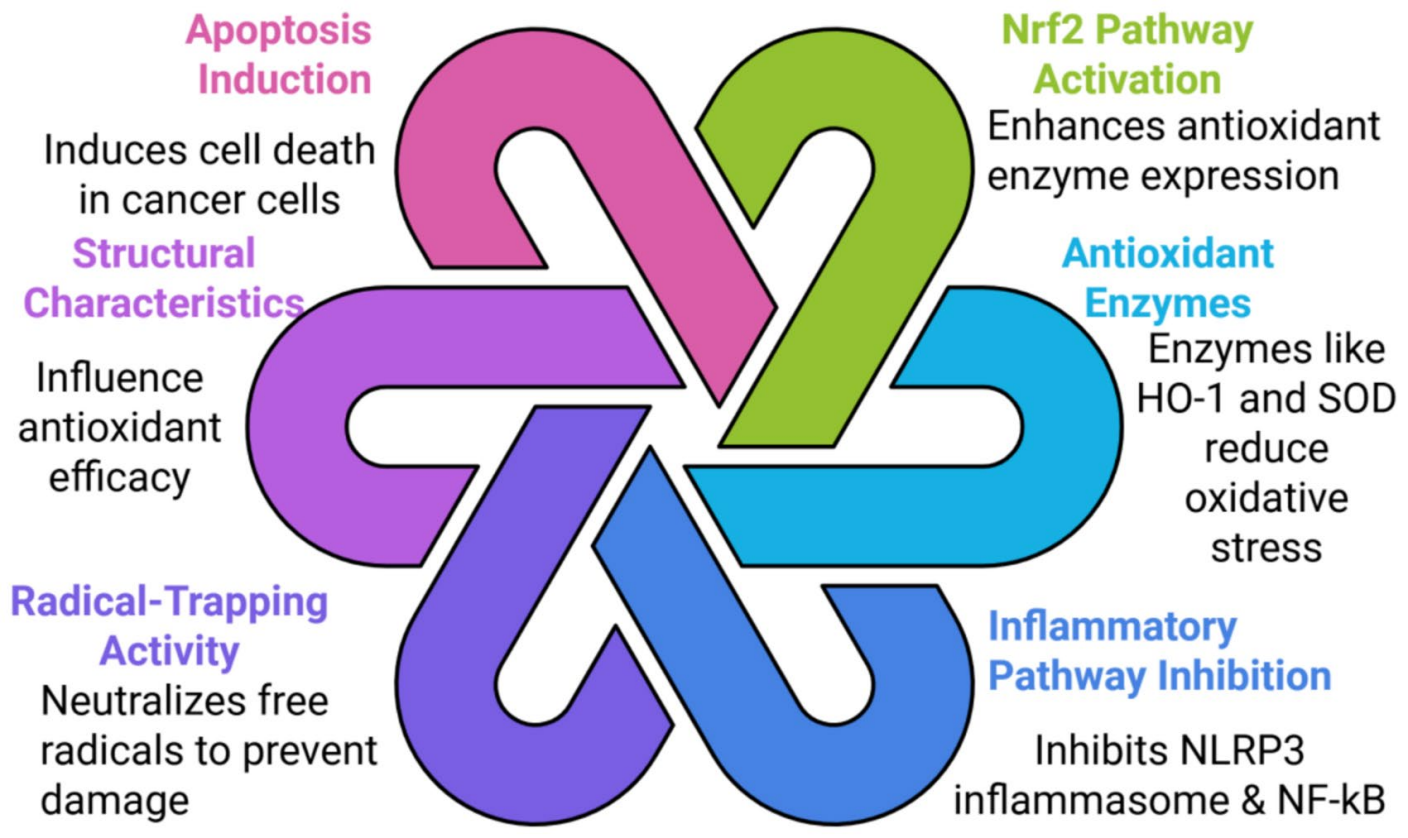
Interlinked molecular mechanisms underlying the pharmacological effects of bioactive compounds. This diagram illustrates the six interconnected molecular pathways through which a bioactive compound (or class of compounds) exerts its therapeutic effects. The six mechanisms are grouped and detailed as follows: Nrf2 Pathway Activation (Green): This mechanism involves the activation of the nuclear factor erythroid 2-related factor 2 (Nrf2) pathway, which serves to enhance antioxidant enzyme expression and bolster the cell’s defense system. Antioxidant Enzymes (Light Blue): The resulting expression of key Antioxidant Enzymes like Heme Oxygenase-1 (HO-1) and Superoxide Dismutase (SOD) effectively reduces oxidative stress within the cell. Inflammatory Pathway Inhibition (Dark Blue): This action involves the direct inhibition of central inflammatory mediators, including the NLRP3 inflammasome and the Nuclear factor kappa-light-chain-enhancer of activated B cells pathway, thereby controlling cellular inflammation. Radical-Trapping Activity (Purple): The compound exhibits direct Radical-Trapping Activity by rapidly neutralizing free radicals in the environment to prevent molecular damage to lipids, proteins, and DNA. Structural Characteristics (Light Purple): The inherent Structural Characteristics of the compound, such as functional groups and molecular geometry, significantly influence its antioxidant efficacy and overall bioactivity. Apoptosis Induction (Pink): A key anti-proliferative mechanism involving the Induction of Apoptosis (programmed cell death) specifically in cancer cells, leading to tumor growth inhibition. These six pathways are depicted as interlinking loops, emphasizing the synergistic and cooperative nature of these molecular actions in promoting overall cellular health and combating disease progression.

The antioxidant activity of allicin involves several intertwined pathways, among which the most crucial one is the induction of the Nrf2 (nuclear factor erythroid 2–related factor 2) pathway, leading to increased expression of antioxidant enzymes such as HO^−1^ (heme oxygenase-1) and SO as shown in Figure 5. Thus, it reduces markers of oxidative stress, which includes MDA (malondialdehyde), and enhances cellular antioxidant capacity. Moreover, it exerts an anti-inflammatory effect by inhibiting the NLRP3 inflammasome and NF-kB, further contributing to its protective effects against oxidative damage (61, 62).

Its key bioactive compounds include allicin and a series of thiosulfinates that have shown radical-trapping antioxidant activity, thus neutralized free radicals and shielding cellular components, including DNA and lipids, from oxidative damage. The structure of the compounds, including the number of sulfur atoms and substitution by particular alk(en)yl groups, also confers the antioxidative efficacy by the potential of such compounds to inhibit low-density lipoproteins (LDL) lipid oxidation (61). In addition, it may cause apoptosis in cancerous cells, inhibition of tumor proliferations, modulation of inflammation, leading to its anti-cancer and anti-inflammatory properties (47, 63). Computational studies have pointed out that allicin and related OSCs scavenge ROS (reactive oxygen species) directly, which enhances their antioxidant efficacy (64). Together, these mechanisms support the potential of allicin for application as a therapeutic agent in pathologies characterized by oxidative stress and inflammation, such as cardiovascular diseases and metabolic syndrome (25).

The structure–activity relationship of OSCs supports that the number of sulfur atoms plays a role in modulating antioxidant potency. Compounds with three sulfur atoms (e.g., DATS) tend to have higher redox potentials and greater ability to donate electrons compared to their disulfide or monosulfide analogs (17, 47). This enables more efficient neutralization of ROS. Moreover, longer alk(en)yl chains increase compound lipophilicity, improving their partitioning into lipid membranes where they exert antioxidant action by scavenging lipid radicals and protecting membrane integrity. These effects are synergistic with their influence on cellular signaling pathways, such as upregulation of antioxidant enzymes through Nrf2 activation (62, 65).

Besides, Allium species have been effective in the management of metabolic syndrome by regulating blood sugar levels, reducing cholesterol, and reducing inflammation, hence lowering cardiovascular risks (8, 29). Their various bioactive roles, inclusive of antimicrobial and anti-inflammatory effects, even constitute their roles in the prevention of chronic diseases: cancer, diabetes, and cardiovascular disorders. Dietary inclusion of Allium species may be contributing toward improved health and various disease conditions (66).

Table 3 showed some bioactive compounds in the Allium vegetables and their various health benefits. Garlic contains a higher amount of allicin, diallyl disulfide, and flavonoids responsible for its antioxidant, anti-inflammatory, and cardio-protective actions. Onions are a rich source of quercetin and sulfur compounds, exhibiting cardiovascular health, immunity, and liver detoxification effects, while leeks contain alliin, allicin, and phenolic acids which may favorably affect heart health and cognitive function (29).

**Table 3:** Bioactive compounds in Allium vegetables and their functions for health.

Allium vegetable	Bioactive compound(s)	Value (approximate)	Health function(s)
Garlic (*Allium sativum*)	Allicin, Diallyl Disulfide, S-allyl-cysteine, Alliin	Allicin content: ~5–10 mg/g in fresh garlic	Antioxidant, anti-inflammatory, antimicrobial, anticancer, improves cholesterol, cardiovascular health
	Quercetin, Kaempferol, Flavonoids	Quercetin: ~50 mg/100 g fresh garlic	Antioxidant, anti-inflammatory, modulates immune response, enhances circulation
Onions (*Allium cepa*)	Quercetin, Kaempferol, Sulfur Compounds (like Diallyl sulfides)	Quercetin: ~30 mg/100 g raw onion	Antioxidant, anti-inflammatory, cardiovascular health, regulates blood pressure, antimicrobial
	S-Alk(en)yl cysteine sulfoxides (SACS)	~3.5–4.0 mg/g (depending on variety)	Anticancer, enhances immunity, supports metabolic health, promotes liver detoxification
Leeks (*Allium ampeloprasum*)	Alliin, Allicin, Quercetin, Phenolic acids	Alliin content: ~6–8 mg/g in raw leek	Antioxidant, anti-inflammatory, antimicrobial, supports heart health and immunity
	Kaempferol, OSCs	Kaempferol: ~0.2 mg/g in leek	Regulates blood pressure, enhances cognitive function, anti-inflammatory
Chives (*Allium schoenoprasum*)	Sulfur compounds (S-allyl cysteine, allicin), Vitamin C	Allicin content: ~10 mg/g in fresh chives	Antioxidant, anti-inflammatory, supports immunity, regulates cholesterol levels, digestive health
	Quercetin, Kaempferol	~0.5 mg/g (varies by cultivation)	Anti-inflammatory, supports cardiovascular health, modulates oxidative stress
Shallots (*Allium cepa var. aggregatum*)	Allicin, OSCs (Diallyl disulfide)	Allicin content: ~8–15 mg/g fresh shallots	Anticancer, antioxidant, cardiovascular health, modulates blood sugar levels, improves gut health

All data in this table are supported by references cited in the main text.

#### 3.1.2 Evidence from clinical and epidemiological studies

Antioxidants from garlic and onion have been pursued in clinical trials for the prevention of chronic diseases. OSCs and flavonoids of garlic and onion have been reported to possess excellent antioxidant, anti-inflammatory, and anti-cancer activities (38). A number of studies have identified compounds with the potential to modulate several signaling pathways implicated in chronic diseases like CVDs (cardiovascular diseases), diabetes, and obesity (16). For instance, bioactive compounds in garlic enhance endothelial function and inhibit lipid peroxidation for vascular protection (27), whereas onion phytomolecules exert a preventive role in non-communicable diseases by acting upon energy metabolism and insulin signaling pathways (38). Clinical evidence has indicated that garlic extracts exhibit antioxidant properties and hence may be of value in the management of chronic diseases like atherosclerosis, diabetes, and hypercholesterolemia. When comparing studies on antioxidant effects, a clear trend emerges: allicin and other OSCs consistently reduce oxidative stress markers such as MDA and increase antioxidant enzyme activity, e.g., SOD (superoxide dismutase), GPx across *in vitro* and *in vivo* models. For example, *in vitro* studies using garlic extract on endothelial and hepatic cells demonstrated strong radical scavenging and Nrf2 activation. In contrast, *in vivo* models in rats confirmed systemic antioxidant benefits but often required higher doses. Clinical trials, while more limited, show moderate improvement in oxidative stress markers in patients with cardiovascular disease or type 2 diabetes. The variations in observed outcomes may stem from differences in bioavailability, compound stability, dosage, and duration of intervention. Notably, studies using stabilized allicin or encapsulated formulations showed more consistent results than raw garlic preparations (47, 67).

### 3.2 Cardiovascular health and anti-inflammatory effects

Among Allium species, garlic, onion, and leek exhibit distinct yet complementary cardioprotective mechanisms mediated by their characteristic phytochemical profiles. Garlic’s organosulfur compounds (OSCs), particularly allicin, S-allyl cysteine, and diallyl disulfide, reduce serum cholesterol and triglycerides by modulating HMG-CoA reductase activity and enhancing nitric oxide–dependent vasodilation, contributing to blood pressure regulation and improved endothelial function (50). In contrast, onion and leek are rich in flavonoids such as quercetin and kaempferol, which exert cardioprotective effects through antioxidant and anti-inflammatory signaling, including inhibition of NF-κB and suppression of LDL oxidation (18). Transitionally, while OSCs primarily act on lipid metabolism and vascular tone, flavonoids modulate vascular inflammation and oxidative stress, indicating a synergistic relationship when these compounds are co-consumed. Studies report that diets incorporating both garlic and onion extracts enhance HDL levels and reduce atherogenic indices more effectively than single-species interventions, suggesting an additive or potentiating interaction (52, 68). Leek and chive further contribute via mild antihypertensive and vasorelaxant properties attributed to sulfur volatiles and phenolic acids, reinforcing the genus-wide cardiovascular benefits of Allium. This integrative understanding underscores how diverse phytochemical pathways across Allium species collectively sustain cardiovascular homeostasis, justifying their inclusion as functional ingredients in heart-healthy diets (50). Moreover, the presence of OSCs and flavonoids makes Allium species anti-inflammatory, contribute to the management of metabolic syndrome and its related disorders (8, 52). These help reduce inflammation as well as improve mitochondrial functions and maintain blood sugar levels, hence helping prevent the progressions of chronic diseases (26). Therefore, the involvement of Allium species in the diet may be regarded as one of the numerous prophylactic policies against chronic inflammation-related disorders (8, 25).

#### 3.2.1 Role of alliums in modulating inflammatory pathways

A wide variety of active principles present in most of the Allium spices have been reported to influence various inflammatory pathways. One proposed mechanism involves the interaction of Allium-derived compounds with the TRPV1 receptor, which may contribute to the attenuation of LPS-induced oxidative stress and inflammation, as suggested by animal model studies. However, this pathway represents only one of several possible mechanisms. Other well-characterized pathways implicated in the anti-inflammatory effects of Allium species include suppression of NF-κB (nuclear factor kappa-light-chain-enhancer of activated B cells) and MAPK signaling cascades, which regulate the expression of key pro-inflammatory cytokines such as IL-6, IL-1β, and TNF-α. These converging pathways highlight the complex and multi-targeted nature of Allium’s anti-inflammatory action (9, 69).

Key compounds include OSCs, flavonoids, and phenolic compounds, which exhibit anti-inflammatory properties by modulating cytokine levels and inhibiting pro-inflammatory mediators. As such, the compounds obtained from onions, in particular, propyl propane thiosulfinate (PTS) and its metabolite, propyl propane thiosulfonate (PTSO), were shown to decrease the amount of two pro-inflammatory cytokines: IL-6 and IL-8, possibly pointing to their application in inflammation treatment. Besides that, polysaccharides isolated from Allium tenuissimum could be potential candidates for ameliorating ulcerative colitis by suppressing the TLR4 (toll-like receptor 4)/MyD88/NF-κB pathway, regulating gut microbiota, and inflammatory cytokines levels accordingly (70, 71).

Moreover, onion’s compound, propyl propane thiosulfinate, has been shown to be anti-inflammatory, as evidenced by the reduction in cytokine levels in many tumor cells. Collectively, this shows that Alliums are potentially useful therapeutics for the modulation of inflammation and associated pathologies (68). Further, onion phytomolecules modulate diverse metabolic pathways, including the processes involved in adipogenesis and insulin signaling, with potential preventive properties against obesity and diabetes. Alliums exert potent anti-inflammatory effects mediated via inhibition of essential inflammatory pathways, such as the HIF-1α pathway, which is implicated with sepsis and chronic inflammation (72). Garlic chive-derived vesicle-like nanoparticles (GC-VLNs) exert potent anti-NLRP3 inflammasome activity, suppressing its downstream pathways, such as caspase-1 autocleavage and cytokine release (73–75).

Allium hookeri demonstrates anti-inflammatory action through the inhibition of the proinflammatory cytokines IL-1β, IL-6, IL-13, and TNF-α in a carrageenan-induced air pouch model, which could be attributed to linoleic acid that acts as a potential candidate for anti-inflammatory drug development (74). These dietary organic sulfur compounds of the species Allium, namely, DADS, DMDS, and PDS, significantly repressed NO production and PGE₂ levels and iNOS and COX-2 mRNA expressions (44, 76). Therefore, acting as effective modulators of the inflammatory pathway. Moreover, intake of the species Allium is related to reduced chronic disease risks, therefore being potentially useful dietary interventions in inflammation management and its related diseases. These collectively propose mechanisms whereby Allium vegetables may assume a therapeutic role in the management of chronic diseases through targeting key inflammatory pathways and metabolic dysregulations (77).

#### 3.2.2 Potential implications for chronic diseases (e.g., cardiovascular diseases, arthritis)

The Allium genus is of considerable importance for managing chronic diseases such as CVDs and arthritis by a number of bioactive compounds in general, but with special emphasis on garlic and onion. One major constituent of garlic, allicin, showed cardioprotection by mitigating the most important processes of diseases like atherosclerosis and hypertension: namely, oxidative stress, inflammation, and apoptosis (50). Additionally, anti-inflammatory and antioxidant activities enabling the latter to support metabolic health and possibly reduce the risk of metabolic syndrome have been made possible by OSCs, flavonoids, and phenolics high in species of Allium (8). Besides, some onion phytomolecules have been associated with antiobesity and antidiabetic activities, an implication of a multichannel approach in the prevention of noncommunicable diseases (38). The OSCsin Allium species have also been reported for the prevention of chronic diseases, including but not limited to cancer, cardiovascular diseases, neurological disorders, diabetes, liver diseases, allergy, and arthritis based on their antibacterial and antioxidant activities besides a proposed mechanism of action (29). Garlic has shown potential pharmacotherapeutic use for life chronic ailments, especially cardiovascular diseases and arthritis, based on its antioxidant, anti-inflammatory, and immunomodulatory activities, which may be useful in the management of conditions such as hypertension, rheumatism, and other inflammatory conditions (6, 78). These findings point toward the therapeutic potential of Allium in the management of chronic ailments, thus justifying further clinical exploration (36).

### 3.3 Antimicrobial activity

Members of the genus Allium, especially garlic and onion, possess very good antimicrobial activities, especially because of their rich phytochemical content, including OSCs like allicin, ajoene, and diallyl sulfides (37, 44, 79). An enhanced antimicrobial action is provided through a combination of extracts obtained from different Allium spp. The combinations sometimes even outperform traditional antibiotics (66, 80). In addition, studies on Ghanaian Allium species proved their prospects for fighting multiresistant infections such as tuberculosis by the mechanism of hindering biofilm development and efflux pump activity In general, the antimicrobial activities of Allium species emphasize their value as pharmaceutical plants in the struggle against microbial resistance and infections (3).

The antimicrobial efficacy of *Allium* compounds, particularly allicin and its breakdown products (such as diallyl polysulfides and ajoenes), is broad-spectrum, targeting Gram-positive and Gram-negative bacteria, fungi, and parasites. This activity is quantifiable through metrics like the Minimal Inhibitory Concentration (MIC) and inhibition zone diameter as mentioned in Table 4.

**Table 4:** Minimal Inhibitory Concentrations (MIC) and pathogen specificity of key *Allium* antimicrobial compounds.

Compound	Target pathogen	MIC value	Specificity/notes
Allicin	*E. coli*	0.125–20 μg/mL	Effective against fermentative bacteria.
Allicin	*S. aureus*	5–10 μg/mL	Effective against Gram-positive cocci.
Allicin	*P. aeruginosa*	20–80 μg/mL	Less potent against non-fermentative bacteria.
Z-Ajoene	Gram-positive bacteria	5–20 μg/mL	Generally more active against Gram-positive strains.
Z-Ajoene	Gram-negative bacteria	100–160 μg/mL	Requires higher concentration for Gram-negative strains.
DADS (Diallyl Disulfide)	*C. albicans* (Yeast)	20–40 μg/mL	Also effective against yeasts.

Furthermore, studies using allicin vapor against lung-pathogenic bacteria, including *Pseudomonas aeruginosa* and various Streptococcus species, demonstrated significant inhibition zone diameters, suggesting potent, concentration-dependent activity.

#### 3.3.1 Mechanisms of action (MoA)

The potent antimicrobial action of Allium compounds stems from their sulfur-containing functional groups and interaction with key microbial processes.

*Allicin and Thiol Groups:* Allicin’s primary bactericidal mechanism is the S-allylmercapto modification of free thiol-containing proteins (cysteine residues) in bacterial cells. This oxidative reaction forms a mixed disulfide bond, which irreversibly inactivates crucial bacterial enzymes. This leads to catastrophic events within the cell, such as the depletion of the internal glutathione pool, induction of protein aggregation, and the inactivation of key metabolic enzymes necessary for survival and electron flow (81).

*Ajoene and Quorum Sensing (QS):* Ajoene and related diallyl polysulfides (such as diallyl disulfide (DAS)) are known to interfere with the bacterial quorum sensing (QS) system. QS is a cell-to-cell communication system that regulates group behaviors like the production of virulence factors and biofilm formation. By disrupting this communication, ajoene inhibits the formation of biofilms, a major factor in chronic and resistant infections, and suppresses the production of virulence factors in pathogens like *Pseudomonas aeruginosa* (82, 83).

#### 3.3.2 *In vitro* and *in vivo* studies on the antimicrobial effects of Alliums

Different antimicrobial activities of the Allium species were impressively enlisted within *in vitro* and *in vivo* studies, especially referring to garlic. It was identified that garlic extracts exhibited high potency against bacteria, including *Staphylococcus aureus*-MRSA, and a fungus, *Candida albicans*, showing a zone of inhibition within a range of 15–35 mm depending on the concentration of the extract (46). The antimicrobial properties are largely attributed to bioactive compounds such as allicin and thiosulfinates, which are present in high concentrations in these extracts. Additionally, studies have shown that chitosan nanoparticles can enhance the antimicrobial efficacy of Allium extracts, particularly against *Mycobacterium tuberculosis*, suggesting a synergistic effect when combined with traditional treatments (27, 47).

Previously, scientists reviewed 42 selected articles, including 1 *in vivo* and 41 *in vitro* studies, they concluded that the antimicrobial properties of the Allium genus essential oils that hadan effect on several microbial species. Evidence proved that these essential oils were multi-potential drugs for their safety and efficacy (45). For instance, some of these OSCs, such as allicin and derivatives like propyl propane thiosulfinate (PTS), exhibit synergistic action against microbes, reaching reductions in bacterial proliferation as high as 99% in ready-to-eat food models (84). Besides this, some aqueous extracts from garlic have been effective against *E. coli* and *C. albicans* clinical isolates. These meta-analyses indeed show that Allium extracts inhibit the growth of pathogens such as *Listeria monocytogenes* and *Staphylococcus aureus* and hence are a promising candidate for food safety biopreservation strategies. The use of Allium species in therapeutic practices has opened a bright perspective in the fight against microbial resistance and enhanced effectiveness of therapy (45).

#### 3.3.3 Mechanisms by which Alliums affect blood pressure and cholesterol levels

Various Allium species, mainly garlic, and onions, have also shown substantial impacts on blood pressure and cholesterol levels, factors of cardiovascular health, via multiple mechanisms as shown in Table 5. Evidence suggests that garlic can lower both systolic and diastolic blood pressures of subjects with hypertension, with apparent effects on the improvement of lipid profiles and reductions in LDL and total cholesterol (85, 86). A systematic review showed that onion supplementation is further helpful in improving metabolic factors, as changes in fat percentages and improvements in the cholesterol level were seen to occur. Flavonoids, particularly flavonols, present in onion, might demonstrate hypolipidemic properties, cardio protection by enhancing endothelial function, hence causing the lowering of blood pressure. These studies have seen the effect of sulfur compounds in garlic enhance the release of NO and impede platelet aggregation, hence lowering blood pressure by some 5–7%, reducing total cholesterol concentration by about 10%, with favorable changes in HDL/LDL ratios (87, 88).

**Table 5:** Summarizing the mechanisms by which Allium species affect blood pressure, cholesterol, and cardiovascular health.

Mechanism/aspect	Allium species	Key compounds	Effects on blood pressure and cholesterol
Blood pressure reduction	Garlic (*Allium sativum*)	Sulfur compounds (e.g., allicin)	Lowers systolic/diastolic BP by 5–7%; enhances nitric oxide release.
Lipid profile improvement	Garlic (*Allium sativum*)	OSCs	Reduces total cholesterol by ~10%, lowers LDL, improves HDL/LDL ratios.
Endothelial function	Onions (*Allium cepa*)	Flavonoids (e.g., flavonols, catechin)	Enhances endothelial function, reduces blood pressure.
Lipid metabolism regulation	Various Allium species	OSCs	Promotes LDL reduction and fecal lipid excretion, prevents hyperlipidemia.
Cardiovascular outcomes	Garlic and onions	OSCs, flavonoids	Reduces CVD risk factors (cholesterol, BP, obesity); lowers CVD incidence.

All data in this table are supported by references cited in the main text.

The OSCs in Allium species further produce lipid metabolism that lowers the level of LDL cholesterol, increasing fecal lipid excretion that inhibits conditions of hyperlipidemia (13). Moreover, Allium eriophyllum extracts have shown antihypertensive and lipid-lowering actions through antioxidant mechanisms and sympatholytic activity (46). These different levels of garlic administration in clinical trials have thus shown significant amelioration of the lipid profile and can possibly position it as a natural therapeutic agent against metabolic syndrome as well as in the prevention of cardiovascular diseases (89). Moreover, being a category of phenolic compounds, they are considered to be active antioxidant molecules; their potential for cardiovascular protection was observed with specific types of compounds like catechin. Some OSCs also present in Allium sp. have been associated with the abovementioned beneficial effects, suggesting that they might provide additional benefits in the management of metabolic syndrome and cardiovascular disease risk factors (47, 62, 90). These findings collectively point toward the therapeutic potential of Allium sp. in managing hypertension and dyslipidemia.

#### 3.3.4 Population studies linking Allium consumption with cardiovascular outcomes

In fact, most of the epidemiological studies suggest that the intake of Allium vegetables, particularly garlic, exerts beneficial cardiovascular protective effects. Systematic reviews and meta-analysis present that, statistically, garlic lowers the risk factors for CVD like hypertension, dyslipidemia, and obesity, characterized by a reduction in total cholesterol, LDL-c, and triglycerides, along with increases in HDL-c (87, 91). Further longitudinal studies have supported such findings, showing an inverse relationship between habitual garlic and onion intake and the incidence of CVD and related diseases (92). It is believed that such effects are mediated by the bioactive compounds, such as OSCs in garlic, through mechanisms involving enhanced fibrinolytic activity and decreased platelet aggregation (93). Although the evidence is promising, further clinical studies are needed to identify the underlying mechanisms and optimize dietary recommendations for cardiovascular health (94).

Across the literature, converging evidence supports the cardiovascular benefits of Allium vegetables, especially garlic. In rodent models, allicin and DATS consistently lowered systolic blood pressure and improved lipid profiles, while human clinical studies reported reductions in total cholesterol (~10%) and blood pressure (~5–7%) following garlic supplementation (95). However, discrepancies arise in populations with different baseline diets or health statuses. Some studies using aged garlic extract found more reliable improvements than those using fresh garlic, likely due to enhanced bioavailability and standardization. Furthermore, epidemiological studies generally align in supporting an inverse association between garlic consumption and cardiovascular disease risk, though some variation exists based on geographic and dietary contexts. These patterns underscore the importance of formulation, dosing, and background diet in interpreting study results (96).

### 3.4 Cancer prevention

Consumption of all vegetables of the Allium group, especially garlic and onions, has been related to cancer prevention, for it contains a large amount of bioactive compounds like allicin and OSCs with manifest strong antioxidant and anticancer effects (36). These compounds have been shown to induce apoptosis, inhibit tumor proliferation, and target several cancer hallmarks, including sustained proliferation and evasion of growth suppressors (94). A systematic review and meta-analysis showed that higher consumption of Allium vegetables is associated with a lower risk of non-digestive tract cancers, with an odds ratio of 0.86 for the highest versus lowest consumption (97). However, no significant association of Allium vegetable consumption was found with the risk for total cancer, showing inconsistency in reported evidence (23). Consumption of allium vegetables, more precisely onions and garlic, has been inversely related to the risk of gastric cancer, especially in Asian populations. These pooled odd ratios tend to show a significantly reduced risk that may point to the possible prevention of cancer with the consumption of Allium (98). These are indeed somewhat mixed findings, but the biological activities of Allium compounds include anti-inflammatory and antitumor effects that do support their possible role in cancer prevention (99).

#### 3.4.1 Overview of research linking Allium consumption with reduced cancer risk

Research indicates a significant association between Allium vegetable consumption, particularly garlic, and reduced cancer risk, particularly for digestive system cancers. A study employing Mendelian randomization found that increased garlic intake is linked to a decreased risk of gastric cancer, while onion consumption did not show a statistically significant association with digestive cancers (100). Moreover, a meta-analysis of 25 studies showed that increased intake of Allium vegetables significantly reduces the risk for non-digestive tract cancers, with a summary odds ratio of 0.86 for the highest compared with the lowest consumption (97). The most noticed bioactive components of Allium species include allicin and OSCs, which affect different aspects of cancer, from cell proliferation to apoptosis (94). A meta-analysis of 17 studies demonstrated that high consumption of Allium vegetables-especially garlic and onion-significantly reduces the risk of developing breast cancer, with pooled risk estimates of 0.70, 0.77, and 0.75 for total intake, garlic, and onion, respectively (23). There is also a report that onionscontains more than 400 compounds showing a wide range of pharmacological action that includes anticancer activities. It also points out that most studies are *in vitro*, while *in vivo* and clinical explorations concerning reduced cancer risk are scanty (101). Further confirm the potentiality of Allium vegetables for use as dietary interventions in cancer prevention strategies.

#### 3.4.2 Potential mechanisms involved in cancer prevention

**Figure 6:**
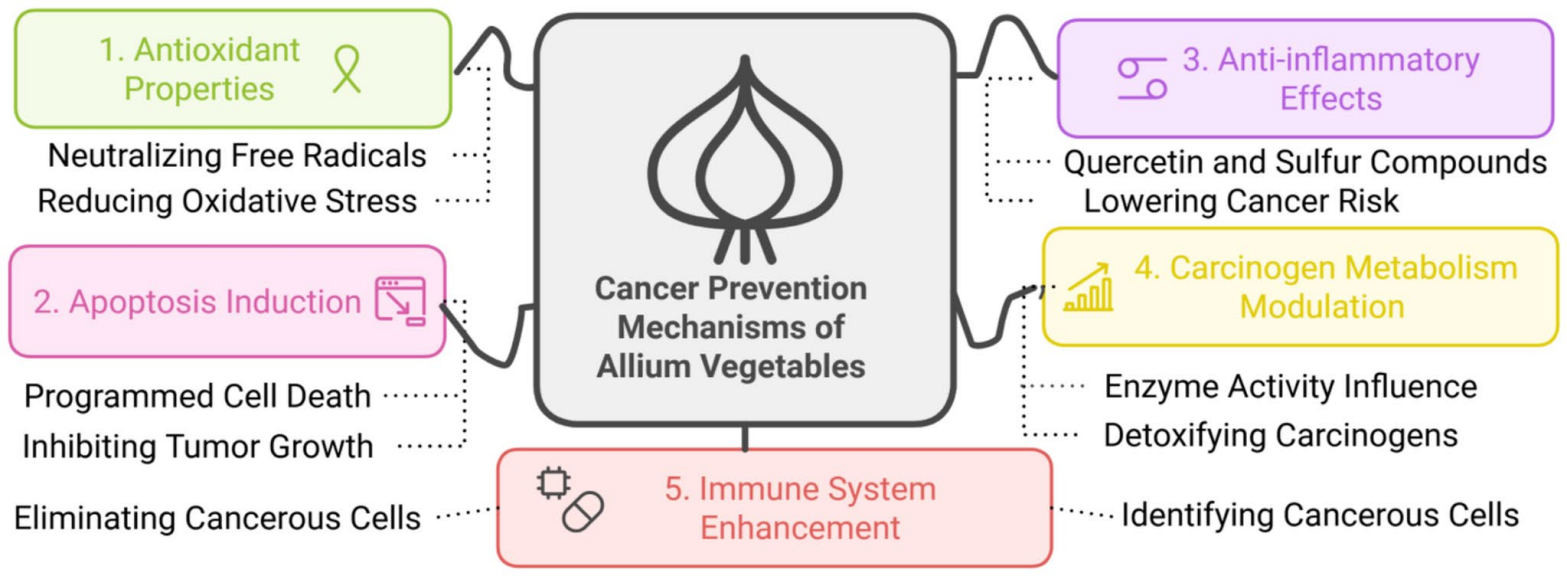
Multifactorial cancer prevention mechanisms of Allium vegetables. This diagram illustrates the five distinct and synergistic pathways through which the bioactive components of Allium Vegetables contribute to Cancer Prevention. The five mechanisms highlighted are: Antioxidant Properties (Green): The compounds neutralize free radicals, effectively reducing oxidative stress. This protective action minimizes the DNA damage that can initiate carcinogenesis. Apoptosis Induction (Pink): Allium compounds trigger programmed cell death in abnormal or cancerous cells. This process, which involves eliminating cancerous cells, is crucial for inhibiting tumor growth and proliferation. Anti-inflammatory Effects (Purple): Attributed specifically to compounds like Quercetin and Sulfur Compounds, these effects mitigate the chronic inflammation known to be a promoter of cancer, thereby lowering cancer risk. Carcinogen Metabolism Modulation (Yellow): The active constituents influence enzyme activity involved in the metabolism of carcinogens. This action enhances the cell’s ability to detoxify carcinogens, rendering them less harmful. Immune System Enhancement (Red): Allium components bolster the body’s natural defenses, assisting the immune system in identifying cancerous cells and initiating an effective clearance response.

The consumption of Allium vegetables, especially garlic and onions, has been associated with several mechanisms that may implicate them in the prevention of cancer (Figure 6). Important bioactive compounds, such as OSCs and flavonoids, have been reported to possess high antioxidant and anticancer activities due to induction of apoptosis, cell cycle arrest, or inhibition of tumor proliferation (102). Certain metabolites, including propyl propane thiosulfinate and propyl propane thiosulfonate, are reported to exert anti-proliferative activity against a wide range of human tumor cell lines, assumed to be at least partially linked to the modulation of oxidative stress and reduction of pro-inflammatory cytokine levels (100). Allium vegetables could exert their anti-cancer properties by induction of apoptosis, mediated by oxidative stress, inhibition of proliferation in tumorigenic cells, reducing the secretion of pro-inflammatory cytokines such as IL-8, IL-6, and IL-17, hence resulting in reduced inflammation and oxidative damage implicated in carcinogenesis (55, 99).

Various cellular pathways inducing cell cycle arrest, promoting apoptosis, and inhibiting angiogenesis are some of the possible methods of cancer prevention induced by principal garlic-derived bioactive compounds, allicin and ajoene, according to animal and *in vitro* studies (103, 104). The anticancer activity of Allium-derived compounds involves multiple molecular targets and signaling pathways. One key mechanism is the induction of apoptosis via the mitochondrial (intrinsic) pathway. Allicin and DATS, for example, have been shown to modulate the expression of pro-apoptotic (e.g., Bax) and anti-apoptotic proteins (e.g., Bcl-2), leading to mitochondrial membrane permeabilization and activation of the caspase cascade, particularly caspase-3 and -9. Additionally, DATS has been reported to upregulate tumor suppressor protein p53 and downregulate cyclin D1, thereby inducing cell cycle arrest at the G2/M phase in breast and prostate cancer cell lines (105, 106).

Differences in compound efficacy have also been reported across cancer types. For instance, DATS shows pronounced activity against breast (MCF-7), prostate (PC-3), and colon (HT-29) cancer cells, while quercetin from onion is more active in lung and pancreatic models. *In vivo* studies in xenograft mouse models confirm tumor volume reduction and increased apoptosis markers after treatment with Allium extracts (107). Some early-phase clinical trials have also shown promising results in reducing cancer biomarkers or lesion growth in colorectal and gastric cancers, though further studies are needed.

## 4 Dietary recommendations and consumption patterns

Current dietary intake recommendations for the family of Allium vegetables consider garlic, onions, leeks, chives, and shallots because of their very high content of bioactive compounds; organosulfur and flavonoids have been associated with a series of health-beneficial properties comprising antioxidant and anti-cancer activities (48).

### 4.1 Recommended intake

The recommendation for these Allium vegetables regarding human diet has been included in the current dietary guidelines, which include garlic and onion for their general health benefits--not only anti-cancerous but also for improving cardiovascular health. A number of epidemiological studies have claimed an association between the regular consumption of these vegetables with a reduced risk of various cancers, including gastric and prostate cancers, with high consumption of Allium-Over 10 g/Day showing significant reduction in the risk of the disease (5). Suggested daily doses that have been advanced in the management of diabetes, cardiovascular diseases, and prevention of cancer, include fresh onion 50 g, fresh onion juice 50 g, and dried onion 20 g (108). Indeed garlic has been found to reduce total cholesterol and improve glycemic control in diabetic patients and hence is a recommended dietary component in patients with dyslipidemia and hypertension. Dietary recommendations of Allium vegetables include daily consumption of 1–3 cloves of raw garlic, which is favorable and creates an enormous positive impact on health due to its various medicinal advantages (109, 110). In Mediterranean diets, the amount of garlic and onions is normally higher, while in Asian diets, shallots and leeks are more common. Overall, inclusion of Allium vegetables in daily diets is suggested for healthy living and avoidance of chronic illnesses (111).

### 4.2 Cultural and culinary uses

The most well-understood representative species are garlic and onion of the genus *Allium*, which has a very important role in both traditional and modern cuisines due to their value in culinary and medicinal purposes (112). Emerging thought leadership underlines the prospects for some underutilized Allium species to further enrich the gastronomic and nutritional profiles of Indian cuisines, such as *Allium tuberosum* (113). Cultivated garlic is used as a condiment and for treating various diseases, while 17% of the investigated population uses the spontaneous *Allium vineale* as a condiment, highlighting their significance in both traditional and modern cuisines (114). With active potential ingredients, alliums have entered the modern diet and cuisine recently when the health view is developing along with the modern trend “food as medicine.” Roasting and boiling improve the bioaccessibility in garlic and onion. As an example, roasting onions increases total polyphenols by 50%, and the bioaccessibility of quercetin increases once taken up with food acidulants such as amchur (115, 116). Further, garlic and onion improve the bioavailability of some essential minerals, namely, iron and zinc from plant-based foods, and their enhancements ranged between 9.4 and 159.4%, depending on a particular food matrix and its preparation method. Onions are grown and consumed throughout the world and are an integral ingredient in most cuisines due to its flavor. In ancient civilizations, it was regarded as one of the most important plants, valued for both medicinal purposes and culinary use, serving to prevent diseases—such as scurvy in ancient Greece—and to enhance the flavor of food (40, 117).

Dietary recommendations, health benefits, and cultural uses of Allium vegetables have been highlighted in Table 6. Garlic is typically consumed in amounts of 1–3 cloves per day and have different values regarding reducing cholesterol, improving glycemic control, and providing antioxidant and anti-cancerous properties, making these vegetables vital components of Mediterranean and Asian cuisines. Onions, with a suggested intake of 50 g fresh or 20 g dried daily, regulate blood pressure and enhance mineral bioavailability, while historically serving culinary and medicinal roles (8). Leeks, rich in sulfur compounds, support heart health and immunity and are prominent in European diets. Chives and shallots, used as garnishes, provide antioxidant benefits and enhance traditional Asian and modern dishes (2, 5, 65).

**Table 6:** Dietary recommendations, health benefits, and cultural uses of Allium vegetables.

Aspect	Details	Recommended intake	Health benefits	Cultural and culinary uses
Garlic (*Allium sativum*)	Contains allicin, OSCs, and phytochemicals.	1–3 cloves daily (fresh garlic).	Lowers cholesterol, improves glycemic control, and exhibits anticancer, antioxidant, and anti-inflammatory properties.	Staple in Mediterranean and Asian cuisines. Used traditionally for medicinal purposes and food preservation.
Onions (*Allium cepa*)	Rich in quercetin, OSCs, and polyphenols.	50 g fresh onion/day or 20 g dried onion/day.	Regulates blood pressure, supports cardiovascular health, and enhances mineral bioavailability.	Used globally for flavor; roasting enhances polyphenol content. Historically used in ancient Greece.
Leeks (*Allium ampeloprasum*)	Contains alliin, sulfur compounds, and phenolic acids.	Consumed widely in Mediterranean diets.	Supports heart health, immunity, and cognitive function.	Popular in European and Asian cooking. Enhances culinary diversity.
Chives (*Allium schoenoprasum*)	Contains vitamin C, sulfur compounds, and flavonoids.	Integrated into diets as garnishes or condiments.	Provides antioxidant benefits, supports immunity, and regulates cholesterol levels.	Enhances flavor; widely used in modern and traditional dishes.
Shallots (*Allium cepa var. aggregatum*)	Rich in OSCs and flavonoids.	Common in Asian and Mediterranean diets.	Exhibits anticancer, antioxidant, and blood sugar-regulating properties.	Widely used in Asian cuisines for their mild flavor and nutritional benefits.

All data in this table are supported by references cited in the main text.

### 4.3 Potential applications of Alliums in food preservation and public health

Edible Allium species, including onion, garlic, leek, chive, and Welsh onion, have been acknowledged for their high values of bioactive compound content, including flavonoids, OSCs, and saponins. These are highly important in health benefits derived from them (35). Besides that, onion waste, which is usually considered worthless, was regarded as a valued source of flavonoids and phenolic compounds because of the various health benefits-total activities as antimicrobial and antioxidant agents. OSCs and steroidal saponins are highly diverse secondary metabolites from this plant, supporting its application in functional foods and its use as natural remedies for chronic diseases. The onion skins were one of the most valued sources for their bioactive compounds: flavonoids, phenolic acids, fructo-oligosaccharides, OSCs, and phenolic glycosides (36, 101). Thus, it can be said that Allium plants have dual applications—as an edible and as antibacterial agents—for possible applications in food biopreservation and health. Hence, Alliums improve not only food safety but also public health thanks to their multitasking biological activities.

## 5 Challenges and future directions

### 5.1 Bioavailability and metabolism

Food processing, chemical interaction, and the food matrix have been mentioned as relevant limitations to the bioavailability of Allium species bioactives, mainly from garlic and onion. Thermal processing significantly alters the chemical composition of Allium-derived bioactives (118). The mode of cooking (e.g., roasting, boiling, frying) and temperature–time profiles play a crucial role in determining the final composition of bioactive compounds, thereby influencing their physiological efficacy (119). Consequently, optimizing preparation methods is essential for preserving or enhancing the health-promoting potential of Allium vegetables. Poor cooking can seriously reduce the amounts of heat-labile OSCs such as allicin and S-allyl cysteine. Besides, their solubility in gastrointestinal fluids and their permeability across the intestinal epithelium surely defines their absorbability. Further research will be conducted on the metabolic routes of different food matrices and the development of new delivery systems with the aim of enhancing their bioavailability (120, 121).

The low bioavailability of Allium-derived bioactives can be attributed to multiple factors. First, many compounds such as allicin and flavonoids exhibit poor aqueous solubility and are chemically unstable in the acidic environment of the stomach. Allicin, for example, rapidly decomposes in the gastrointestinal tract, limiting its absorption in intact form. Second, extensive first-pass metabolism in the liver and intestines, including enzymatic conjugation reactions, reduces the systemic availability of the parent compounds. Third, variability in individual gut microbiota influences the biotransformation and absorption of phenolic and OSCs, leading to inconsistent biological responses across populations (43). To overcome these barriers, several strategies have been proposed and tested. Nanoencapsulation using liposomes, solid lipid nanoparticles, or polysaccharide-based carriers can protect bioactives from degradation and enhance their intestinal absorption (122, 123).

### 5.2 Consumer awareness and acceptance

Barriers to increased consumption include overall lack of awareness about the health benefits of Allium species, cultural preferences, and poor access to fresh Allium products. Although the possible antioxidant and anticancer properties of Allium vegetables may just start to become recognized, few people are aware of what specific health benefits might be attributed to Allium species, for example, their place in metabolic syndrome management and anti-inflammation. Aside from preparation and time, culinary unfamiliarity is bound to further discourage consumption since most are ignorant of how to use them in their diet. Possible promotional strategies for Allium include raising awareness educations on its health benefits, enhancing cooking skills, and giving better access to fresh Allium produce in disadvantaged communities (120). Addressing such barriers and providing targeted strategies may lead to a better realization of the health benefits of Alliums among various populations.

### 5.3 Traditional uses versus current pharmacological findings

Traditionally, Allium species have been consumed both as food and medicine, valued for their ability to “purify the blood,” prevent seasonal illnesses, and treat respiratory or digestive ailments. In many cultures, garlic (*Allium sativum*) was used as a remedy for infections, to boost vitality, and as a cardiovascular tonic, while onions (*Allium cepa*) and leeks (*Allium ampeloprasum var. porrum*) were incorporated for their diuretic and anti-inflammatory properties. Modern pharmacological studies have validated several of these claims, particularly in the areas of antimicrobial, cardioprotective, and antioxidant activity, attributing these effects to OSCs (e.g., allicin, diallyl disulfide) and flavonoids (e.g., quercetin) (6, 124). However, current research has also revealed mechanisms not recognized in traditional medicine, such as modulation of gene expression, inhibition of specific signaling pathways (NF-κB, Nrf2), and targeted effects on metabolic disorders like type 2 diabetes and certain cancers. In contrast, some traditional uses—such as employing Allium as a universal cure or for mystical protection—lack scientific support. This comparison underscores the importance of integrating ethnomedicinal wisdom with rigorous pharmacological evaluation to develop evidence-based functional foods and therapeutics derived from Allium species (116, 125).

### 5.4 Safety, toxicity, and adverse effects

While Allium vegetables are broadly recognized as safe when consumed in dietary amounts, their concentrated supplements can exert dose-dependent physiological effects requiring careful consideration. Clinical and pharmacological studies have demonstrated that culinary doses of garlic (2–5 g fresh cloves per day) or onion (50–100 g/day) are well tolerated and unlikely to produce adverse outcomes. However, high-dose garlic extracts or aged garlic supplements (>10 g/day or >600 mg/day allicin equivalent) have been associated with increased bleeding risk when co-administered with anticoagulants (e.g., warfarin, aspirin) due to potentiation of fibrinolytic and platelet inhibition pathways (95).

Moreover, individuals with liver or kidney dysfunction may exhibit altered metabolism of sulfur compounds, necessitating dose adjustments or medical supervision. Pregnant and lactating women are generally advised to limit supplement use due to insufficient data on high-dose safety, although dietary consumption remains safe. Children and elderly populations may experience greater gastrointestinal sensitivity, emphasizing the need for moderation and professional guidance.

Recent toxicity evaluations confirm a high margin of safety for dietary intake, with an estimated NOAEL (No Observed Adverse Effect Level) exceeding 2 g/kg body weight in animal studies (129). Nonetheless, appropriate labeling and consumer education on supplement dosage and potential drug–nutrient interactions remain critical for public health safety. Dose context. Typical dietary use equals ~1–3 cloves/day of garlic, while clinical studies frequently use aged garlic extract at ~1.2 g/day (providing S-allyl-cysteine), with acceptable safety in trials up to several months; longer-term use has also been reported as safe in research settings (126). Drug–supplement interactions and perioperative care. Garlic can inhibit platelet aggregation and may potentiate the effects of antiplatelet/anticoagulant drugs (e.g., aspirin, warfarin), increasing bleeding risk; many surgical guidelines advise discontinuing garlic supplements prior to elective procedures. Clinicians should be informed of use (116, 127).

Digestive intolerance (FODMAPs). Garlic, onions, leeks, and related Alliums are high in fructans (FODMAPs) and may exacerbate symptoms in individuals with irritable bowel syndrome; dietary management may require limitation or modified preparation methods. Populations requiring caution. Individuals on anticoagulants/antiplatelets, those with known Allium allergy, and patients scheduled for surgery should use supplements cautiously. Topical application of raw garlic should be avoided due to risk of burns (34).

### 5.5 Future research areas

Literature available on Alliums indicates some critical gaps, especially in the research of the underutilized species other than garlic and onions, which monopolized the literature up until now. While most of the current research into the antioxidant and anticancer activities of Allium species and their active principles organosulfur compounds (OSCs) and flavonoids-is promising, much more clinical trials need to be conducted in various populations and environmental conditions to confirm the observations (126). Also, the possible role of Alliums in managing metabolic syndrome and associated disorders is still not well studied, and epidemiological studies to confirm its efficacy and safety are needed (34). Integrating traditional knowledge into a modern scientific approach can progress the understanding and applications of Allium species for health interventions, thus opening new perspectives for the development of new therapeutic strategies (128).

## 6 Conclusion

This comprehensive review has synthesized insights from nutritional science, pharmacology, and clinical evidence to establish the Allium genus as an essential foundation for preventative health. Unlike previous work, we have integrated emerging perspectives—highlighting not only the profound effects of organosulfur compounds (OSCs) and flavonoids but also novel aspects like gut microbiota interaction, bioavailability challenges, and technological innovations for maximizing efficacy. Allium vegetables, including garlic, onions, leeks, and chives, are indispensable components of human diets due to their rich nutritional and therapeutic profiles. These components confer significant antioxidant, anti-inflammatory, and antimicrobial properties, collectively supporting cardiovascular health, regulating metabolism, and aiding in the prevention of chronic diseases. Bioactive molecules such as allicin and quercetin exhibit validated anticancer and chemopreventive effects by reducing oxidative stress and modulating key cellular pathways. Regular consumption, such as 1–3 cloves of garlic or 50 g of onions daily, is strongly linked to reduced risks of cancer and metabolic disorders. Despite these established advantages, challenges remain, particularly concerning the low bioavailability of certain compounds and limited public awareness. Overcoming these obstacles requires innovative delivery systems, targeted public education, and strategic research. The future trajectory of Allium research must therefore focus on bridging traditional knowledge with modern scientific advancements. Traditional systems like Ayurveda and Traditional Chinese Medicine (TCM), which historically used specific preparations (e.g., garlic for respiratory conditions), provide crucial ethnomedicinal guidance for modern inquiry. For instance, the traditional anti-infective use of garlic justifies current clinical trials into its antiviral and immune-modulating effects. Future efforts should prioritize underutilized species, such as *A. victorialis* or *A. tuberosum*, that have rich traditional value but insufficient modern profiling. By coupling genomic analysis and advanced bioavailability studies with methods guided by traditional preparation, we can move beyond the current focus to identify novel, highly potent molecules in the wider Allium family, solidifying their global role as functional foods that promote sustainable health strategies worldwide.
